# Increasing Trends in Hospitalisation With Diabetic Ketoacidosis at Type 1 Diabetes Diagnosis Among Children and Youth in the United States and England, 2002–2020

**DOI:** 10.1111/dom.71279

**Published:** 2026-08-27

**Authors:** Chintan V. Dave, Kristen Lloyd, Abner Nyandege, Jason Roy, Raquel López‐Díez, Sally Radovick, Brian L. Strom

**Affiliations:** ^1^ Center for Pharmacoepidemiology and Treatment Science Institute for Health, Health Care Policy and Aging Research, Rutgers University New Brunswick New Jersey USA; ^2^ Center for Health Outcomes, Policy, and Economics, Ernest Mario School of Pharmacy Rutgers University Piscataway New Jersey USA; ^3^ Department of Biostatistics and Epidemiology Rutgers School of Public Health Piscataway New Jersey USA; ^4^ Breakthrough T1D (Formerly JDRF International) New York New York USA; ^5^ Division of Pediatric Endocrinology The University of Arizona College of Medicine Tucson Tucson Arizona USA; ^6^ Rutgers Biomedical and Health Sciences Newark New Jersey USA

**Keywords:** database research, diabetes complications, pharmaco‐epidemiology, Type 1 diabetes

## Abstract

**Background:**

There is a paucity of population‐level data evaluating trends in the proportion of children and youth hospitalised with diabetic ketoacidosis (DKA) at the time of Type 1 diabetes (T1D) diagnosis.

**Methods:**

National Medicaid (US publicly insured claims), MarketScan (US commercially insured claims) and Clinical Practice Research Datalink (CPRD) (English electronic health record) data were used to create cohorts of children with incident T1D. DKA at diagnosis was defined as a DKA hospitalisation occurring within 30 days prior to the diagnosis of T1D. The likelihood of presenting in DKA was modelled using age, sex, race and community‐level social vulnerability. Population‐level T1D incidence trends were also assessed.

**Results:**

Among 116 845 patients (mean [SD] age: 10.4 [4.9]; 46.8% female), 48 291 (41.3%) presented in DKA. These proportions increased between 2002 and 2019, rising by 11.1% (from 34.4% to 45.5%) in Medicaid, 2.8% (from 42.0% to 44.8%) in MarketScan and 18.6% (from 17.9% to 36.5%) in CPRD, and further increasing in 2020 (Medicaid, 52.4%; MarketScan, 52.9%; CPRD, 44.1%). T1D incidence increased from 2002 to 2019 from 10 to 17, 12 to 20 and 5 to 13 per 100 000 children, respectively, reaching 21, 22 and 15 per 100 000 in 2020, respectively. DKA was higher among children aged 6 months to 2 years (70.4%), and among Black (45.4%) and Hispanic (45.4%) backgrounds. An increased relative risk of DKA was evident among children under 2 years: 1.97 (95% CI: 1.92–2.02), Black children: 1.14 (1.06–1.23), Hispanic children: 1.12 (1.10–1.15), and those residing in higher areas of social vulnerability: 1.20 (1.02–1.40).

**Conclusion:**

This study found a relative increase in the proportion of children presenting with DKA at T1D diagnosis, occurring in parallel with rising T1D incidence. This uptrend, which preceded but was exacerbated by the COVID‐19 pandemic, underscores worsening epidemiological trends, especially notable in England and among publicly insured US children.

## 1 Introduction

Diabetic ketoacidosis (DKA)—a metabolic derangement characterised by the triad of hyperglycemia, metabolic acidosis and ketosis—is a leading cause of hospital admissions, morbidity and mortality among children with Type 1 diabetes (T1D) [1, 2, 3]. Despite aggressive management, children with DKA can develop life‐threatening complications, including pancreatic injury, acute respiratory distress, as well as cerebral oedema [2, 3, 4, 5]. DKA is also often the presenting symptom of children newly diagnosed with T1D [6, 7]. Children who present in DKA often exhibit a less favourable long‐term clinical course, characterised by an accelerated decline in β‐cell function [8], diminished likelihood of disease remission [9], suboptimal long‐term glycaemic control and higher risk of T1D complications [10, 11].

Despite the significant health consequences associated with hospitalisation for DKA at T1D diagnosis (‘DKA at diagnosis’), there is a paucity of nationally representative data evaluating these trends. The clinical utility of prior studies is limited due to short time horizons—precluding assessment of long‐term trends [12, 13, 14, 15], reliance on single centre settings—reducing the generalizability of findings, and small sample sizes—which limit the overall power as well as ability to examine pertinent subgroups [12, 13, 14, 16]. Further, estimates of DKA derived from prospective cohorts of children at risk of T1D, such as the Diabetes Autoimmunity Study in the Young (DAISY), may not reflect the general population. For instance, the rates of DKA at diagnosis were substantially lower in the DAISY (3%) compared with the non‐DAISY (44%) group [17]. Finally, it is unclear whether the improvements in medical care coupled with the rising awareness of earlier detection of T1D have resulted in corresponding declines in the national rates of DKA at diagnosis, especially when contextualised against the global backdrop of rising T1D incidence [18]. Taken together, these factors limit our ability to draw inferences regarding longitudinal trends in the general population, underscoring the need for more robust, population‐based studies capturing real‐world paediatric populations rather than selected high‐risk cohorts.

Accordingly, the aim of this study was to leverage large, nationally representative data encompassing commercially and publicly insured children and youth in the United States and population‐based electronic health record (EHR) data from England to quantify long‐term trends in the proportion of patients hospitalised with DKA at diagnosis (‘DKA at diagnosis’). We further characterised these trends across subgroups defined by age, sex and social vulnerability, and estimated the associated relative risks. Finally, we evaluated the corresponding temporal trends in the incidence of T1D within each dataset.

## 2 Methods

This study was approved by the Rutgers University Institutional Review Board and the requirement for informed consent was waived. Appropriate data use agreements were in place.

### 2.1 Data Sources

We used two population‐based datasets from the United States and one from England spanning from 1 January 2002, to 31 December 2020. From the United States, the Medicaid Analytic eXtract‐Transformed Medicaid Statistical Information System Analytic Files (MAX‐TAF) (‘Medicaid’) and Merative MarketScan Database (‘MarketScan’) were utilised. These data provide information on US Medicaid beneficiaries and commercially insured children, respectively. Both claims datasets contain information on patient demographics, inpatient and outpatient diagnosis codes (coded using the International Classification of Diseases Ninth and Tenth Revision, Clinical Modification), outpatient pharmacy dispensing (coded using National Drug Codes) and patient enrolment files. Using the patient's county of residence, Medicaid data were linked to the Social Vulnerability Index (SVI) from 2010 onward, coinciding with the SVI's availability [19]. MarketScan data lacked the requisite granularity of geographical information for a similar linkage.

From England, the Clinical Practice Research Datalink Gold and Aurum data (‘CPRD’) were used. CPRD is an integrated, longitudinal database containing EHR of patients sourced from > 1100 practices throughout England [20]. Medical information—including patient symptoms, diagnoses and lifestyle measures (e.g., smoking status)—is recorded using Read codes (version 2) by the general practice staff during consultations. Prescriptions issued by the provider are captured in the database along with the product name and British National Formulary Codes. For patients residing in England, information on hospitalisation and social deprivation is available through linkages with the Hospital Episode Statistics files and multiple deprivation files, respectively [20].

### 2.2 Study Population

In each dataset, a cohort of children aged 6 months to 21 years newly diagnosed with T1D was identified, serving as the study denominator for the primary analysis. Individuals were classified as having incident T1D if a first‐ever diagnosis of T1D was recorded from 30 days prior to 3 days following their first‐ever prescription for insulin. The date of the insulin prescription was designated as the index date, and patients were required to be continuously eligible in the data for a 6‐month period prior to the index date. This approach is associated with a positive predictive value (PPV) exceeding 94% in identifying T1D [21].

The 6 month period prior to the index date was classified as the baseline period, during which all eligibility criteria and other baseline characteristics were ascertained. Cohort membership was further restricted to children who were continuously eligible during the baseline period, and to those who had no evidence of other forms of diabetes (e.g., Type 2 diabetes) or use of glucose lowering therapies. Infants diagnosed with T1D within the first 6 months of life were excluded to permit a 6‐month baseline lookback period.

### 2.3 DKA at Diagnosis and Other Patient Characteristics

Patients were classified as having DKA at diagnosis if they had evidence of a hospitalisation with corresponding codes for DKA within 30 days prior to the index date (PPV > 88%) [22]. No clinical or biochemical criteria, including pH, bicarbonate, anion gap or serum ketone measurements, were available or used to define DKA.

Race and ethnicity were ascertained from self‐reported information recorded in each data source. In Medicaid, race and ethnicity were obtained from enrolment files, including MAX and TAF. In CPRD, ethnicity was obtained from primary care records and linked HES data, when available. These data were harmonised into a single mutually exclusive categorical variable comprising non‐Hispanic White (‘White’), non‐Hispanic Black (‘Black’), Hispanic, Asian, Other and unknown or missing. In CPRD, Hispanic ethnicity was grouped with Other because the small number of Hispanic children precluded separate assessment. This single harmonised variable was included in the regression models rather than separate race and ethnicity variables.

Additional patient characteristics, including the age at index date and sex, were also assessed. Area‐level social determinants of health were ascertained using the SVI (available from 2010 onwards) for Medicaid [19], and the Index of Multiple Deprivation for CPRD [20].

### 2.4 Statistical Analysis

All analyses were performed using SAS 9.4 (SAS Institute Inc., Cary, NC). The study period was segmented into calendar year intervals, and each initiation episode was assigned to a calendar year according to the index date. The primary analysis assessed the proportion of newly diagnosed T1D cases presenting with DKA, calculated as the number of patients with DKA at diagnosis (numerator) divided by all incident T1D cases (denominator). Temporal trends in DKA at diagnosis were evaluated visually through graphical presentation and statistically using regression models to assess for the presence of linear secular trends. Trends were evaluated both through 2019 to characterise pre‐2020 patterns, and through 2020 to assess whether overall temporal trends were affected by inclusion of 2020.

Due to the relatively high rate of DKA at diagnosis, modified Poisson regression models were estimated to elucidate relative risks, with DKA at diagnosis as the dependent variable and sex, age, race, ethnicity, calendar year and social vulnerability as the independent variables. Overall estimates pooled across databases were derived from random effects meta‐analysis [23].

We then conducted three analyses. First, we estimated the incidence of T1D across the three databases by calendar year. This analysis sought to explore whether any observed trends in DKA at diagnosis corresponded to the changes in the underlying rates of T1D. For this analysis, the denominator included children with at least 6 months of continuous enrolment during the year and no prior evidence of T1D (diagnosis codes or insulin use), and the numerator comprised incident T1D cases. Second, we conducted a sensitivity analysis to evaluate the robustness of our case‐identification algorithm. In this analysis, we modified the definition to require incident insulin use without a concurrent T1D diagnosis, acknowledging that T1D may not always be coded in a timely manner. Third, we evaluated trends in DKA at diagnosis across pertinent subgroups of age (6 months to < 2, 2 to < 7, 7–13 and > 13), sex, race and ethnicity, and degree of social vulnerability.

## 3 Results

After the eligibility criteria were applied, we identified 81 561 patients newly diagnosed with T1D in Medicaid, 24 176 in MarketScan and 11 108 in CPRD (see Supporting Information Appendix Figure 1 for CONSORT flow diagrams). The total pooled cohort was comprised of 116 845 patients, of whom 48 921 (41.3%) presented in DKA (see Supporting Information Appendix Table 1 for patient characteristics by individual database). The overall proportion of DKA at diagnosis was 42.6% in Medicaid (publicly insured US children), 42.9% in MarketScan (commercially insured US children) and 28.8% in CPRD (English children) (Supporting Information Appendix Table 1).

The proportion of DKA at diagnosis was highest among children aged 6 months to 2 years (70.4%) and decreased with increasing age. Higher proportions were observed among Black (45.4%) and Hispanic (45.4%) compared with White (37.8%) children, among those living in areas of high versus low social vulnerability, and in later compared with earlier calendar years (Table 1).

**TABLE 1:** Pooled characteristics of patients newly diagnosed with T1D in the United States and England (2002–2020).^a^

	Overall cohort (*n* = 116 845)	With DKA at diagnosis (*n* = 48 291)	Without DKA at diagnosis (*n* = 68 554)	SD^b^	Proportion of subgroup with DKA at diagnosis
Sociodemographics					
Age, mean (StD), years	10.4 (4.9)	10.0 (5.1)	10.7 (4.8)	14.1	
6 months < 2 years	3517 (3.0)	2475 (5.1)	1042 (1.5)	20.3	70.4%
2 to < 7 years	24 054 (20.6)	10 078 (20.9)	13 976 (20.4)	1.2	41.9%
7–13 years	57 390 (49.1)	23 969 (49.6)	33 421 (48.8)	1.6	41.8%
> 13 years	31 884 (27.3)	11 769 (24.4)	20 115 (29.3)	11.1	36.9%
Male	62 160 (53.2)	26 202 (54.3)	35 958 (52.5)	3.6	42.2%
Female	54 683 (46.8)	22 087 (45.7)	32 596 (47.5)	3.6	40.4%
Race and ethnicity^c^					
White	44 883 (48.4)	16 957 (44.7)	27 926 (51.0)	12.6	37.8%
Black	17 996 (19.4)	8173 (21.6)	9823 (17.9)	−9.3	45.4%
Hispanic	14 113 (15.2)	6413 (16.9)	7700 (14.1)	7.7	45.4%
Asian	1638 (1.8)	640 (1.7)	998 (1.8)	0.8	39.1%
Other	14 039 (15.2)	5744 (15.1)	8295 (15.2)	0.3	40.9%
Calendar year					
2002–2005	13 157 (11.3)	4707 (9.8)	8450 (12.3)	8.0	35.8%
2006–2009	18 209 (15.6)	6783 (14.1)	11 426 (16.7)	7.2	37.3%
2010–2015	42 027 (36.0)	16 782 (34.7)	25 245 (36.8)	4.4	39.9%
2016–2019	33 201 (28.4)	14 703 (30.5)	18 498 (27.0)	7.7	44.3%
2020	10 251 (8.8)	5316 (11.0)	4935 (7.2)	13.2	51.9%
Social vulnerability^c^					
Lowest tercile	23 156 (33.6)	9084 (31.6)	14 072 (35.0)	7.2	39.2%
Middle tercile	22 440 (32.6)	9139 (31.8)	13 301 (33.1)	2.8	40.7%
Highest tercile	23 333 (33.8)	10 523 (36.6)	12 810 (31.9)	9.9	45.1%

Abbreviations: SD: standardised differences; StD: standard deviation; T1D: Type 1 diabetes.

^a^ Pooled characteristics are shown across Medicaid (United States), MarketScan (United States) and CPRD (England) data. See Supporting Information Appendix Table 1 for database specific characteristics.

^b^ Values of > 10% indicate a meaningful difference.

^c^ Information on race, ethnicity and community‐level social vulnerability were available for Medicaid (after 2010 for social vulnerability) and CPRD, but not MarketScan. The denominator for race/ethnicity estimates was 92 669 (37 927 with DKA, 54742 without). The denominator for social vulnerability estimates was 68 929 (28 746 with DKA and 40 183 without).

### 3.1 Overall Trends in DKA at Diagnosis and Incidence of T1D

Figure 1A delineates the trends in the proportion of children presenting with DKA (see Supporting Information Appendix Table 2 for additional details). In Medicaid, this proportion increased by 11.1% from 34.4% in 2002 to 45.5% in 2019, reaching 52.4% in 2020 (*p* < 0.001 for trend). In MarketScan, the proportion declined slightly through 2010 before increasing to 44.8% in 2019 and 52.9% in 2020 (*p* < 0.001). In CPRD, it more than doubled, increasing from 17.9% in 2002 to 36.5% in 2019 and 44.1% in 2020 (*p* < 0.001). All datasets showed significant upward trends prior to 2020, preceding the pandemic‐associated surge (*p* < 0.01 for trends through 2019 in each database).

**FIGURE 1:**
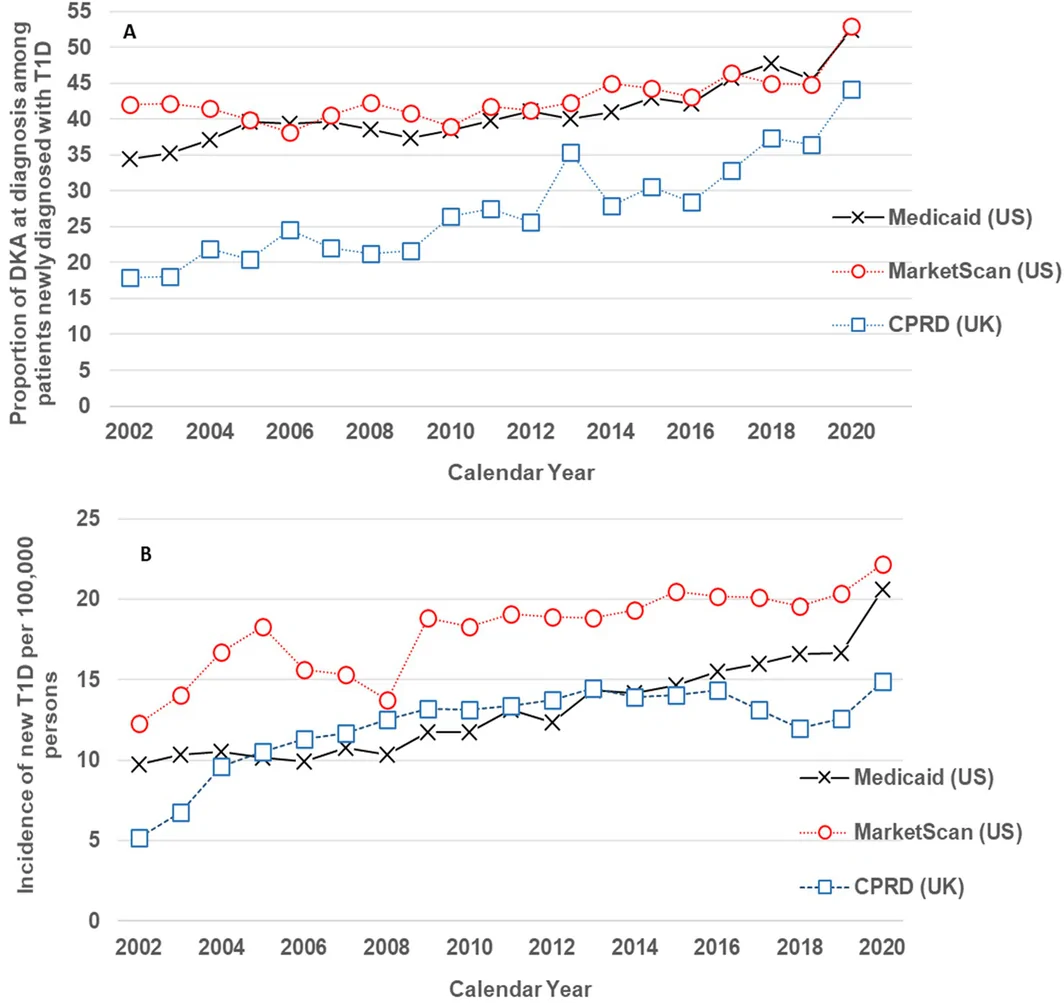
Overall trends in DKA hospitalisations at diagnosis of Type 1 diabetes in Medicaid, MarketScan and CPRD data. CPRD: Clinical Practice Research Datalink; DKA: diabetic ketoacidosis. The figure describes secular trends in (A) the proportion of newly diagnosed children with T1D presenting in DKA and (B) the incidence of T1D. See Supporting Information Appendix Table 2 for additional details.

Figure 1B depicts the trends in the incidence of T1D. In Medicaid, the incidence was stable through 2008, increased from 10 to 17 cases per 100 000 in 2019, and 21 in 2020 (*p* < 0.001 for trend). In MarketScan, T1D incidence increased from 12 cases per 100 000 in 2002 to 20 in 2019 and 22 in 2020, whilst in CPRD it increased from 5 cases per 100 000 in 2002 to 13 in 2019 and 15 in 2020 (*p* < 0.001 for trends).

### 3.2 Subgroup and Sensitivity Analyses

Among males, DKA proportions increased through 2019 by 12.4% in Medicaid, 1.2% in MarketScan and 21.5% in CPRD (Figure 2 and Supporting Information Appendix Table 3), with further increases in 2020 of 7.2%, 7.3% and 13.4%, respectively. Among females, corresponding increases through 2019 were 9.4%, 4.6% and 15.3%, with further increases in 2020 in Medicaid and MarketScan but little change in CPRD. In children younger than 2, for whom estimates were pooled for 2–3 years due to sample size, DKA proportions changed by −0.8% in Medicaid, 14.1% in MarketScan and 35.5% in CPRD; none of the trends were statistically significant, with small sample sizes yielding imprecise estimates and limited power. In children 2 to younger than 7 years, increases through 2019 ranged from 8.1% to 13.6%, whilst among those older than 13 years, increases ranged from 1.2% to 25.7% across databases; most groups increased further in 2020. In Medicaid, DKA proportions increased among non‐White and White patients through 2019 by 10.1% and 11.4%, respectively, with further increases in 2020 (Figure 3 and Supporting Information Appendix Table 4). In CPRD, increases through 2019 were more pronounced among White patients, with both groups increasing further in 2020. Differences by social vulnerability were modest in Medicaid but more pronounced in CPRD.

**FIGURE 2:**
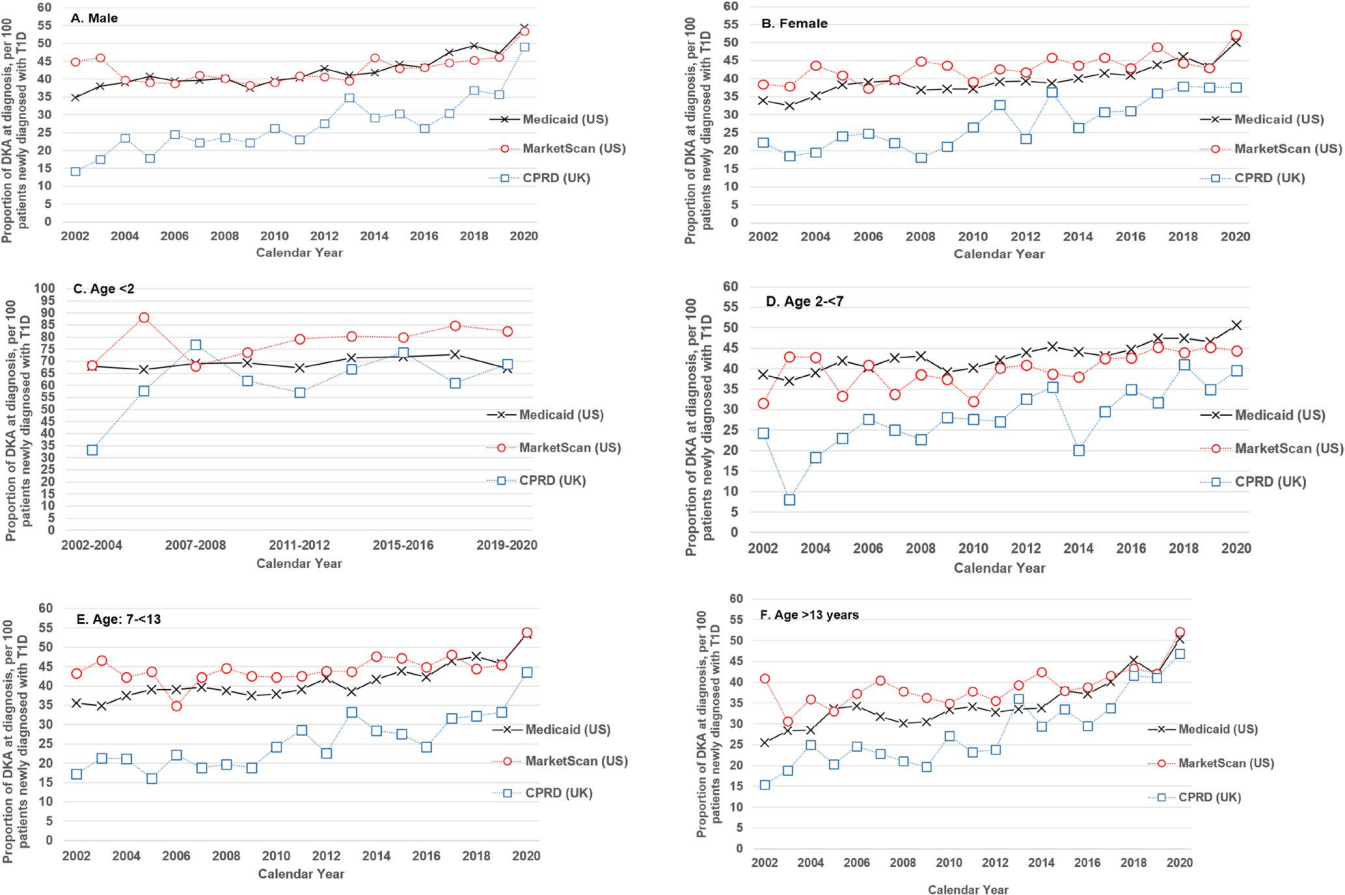
Trends in DKA hospitalisations at diagnosis of Type 1 diabetes, by subgroups of age and sex. CPRD: Clinical Practice Research Datalink; DKA: diabetic ketoacidosis. The figure describes secular trends in the proportion of newly diagnosed Type 1 diabetes children presenting in DKA at diagnosis, by sex and age.

**FIGURE 3:**
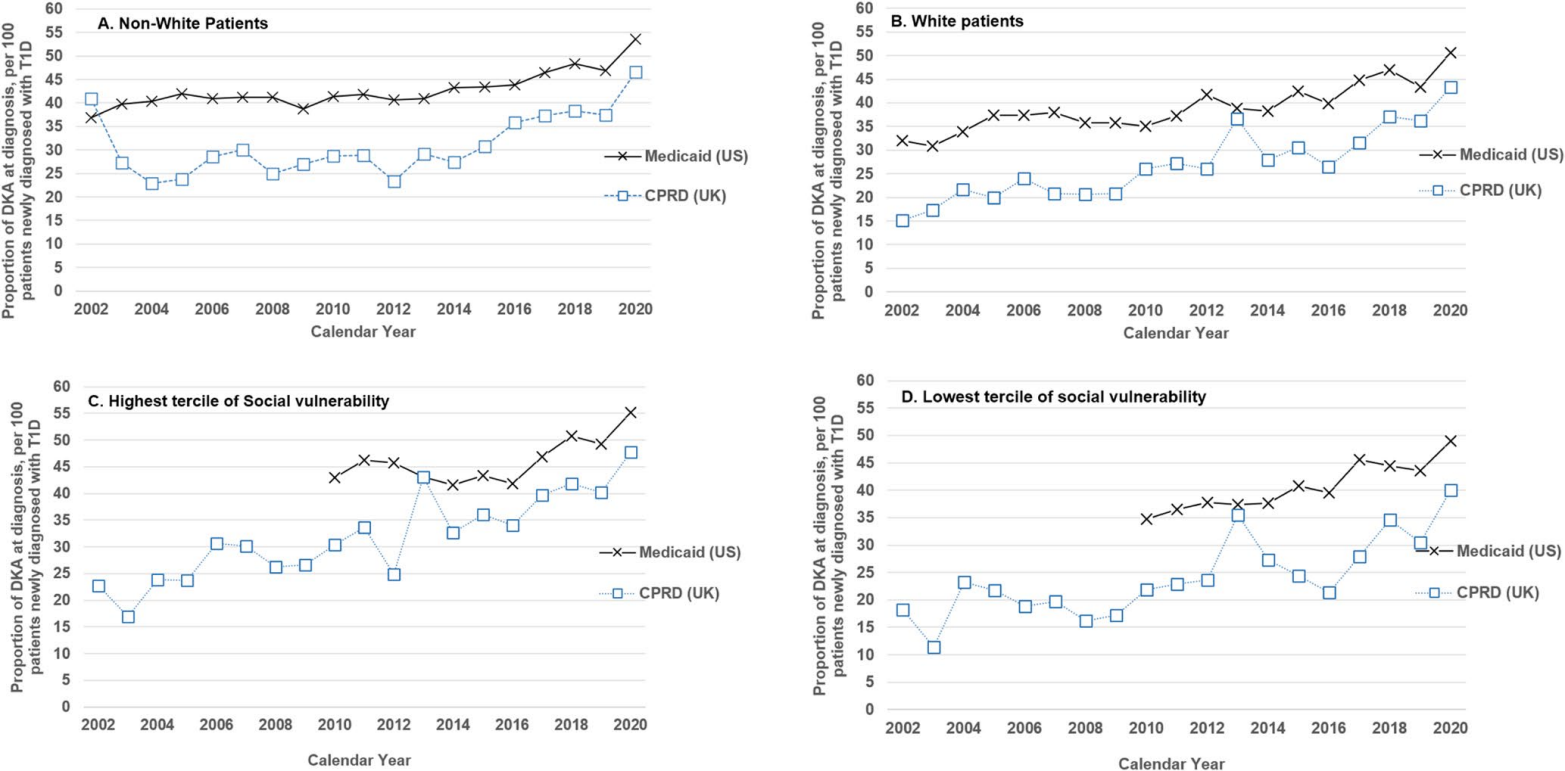
Trends in DKA hospitalisations at diagnosis of Type 1 diabetes, by race and community level social vulnerability. CPRD: Clinical Practice Research Datalink; DKA: diabetic ketoacidosis. The figure describes secular trends in the proportion of newly diagnosed Type 1 diabetes patients presenting in DKA at diagnosis, by race and community level social vulnerability. Data for race and ethnicity were available for Medicaid and CPRD, but not MarketScan. Community level social vulnerability information was available for Medicaid after 2010. See Supporting Information Appendix Table 4 for additional information.

Sensitivity analyses restricted to patients defined solely by insulin initiation (Supporting Information Appendix Table 5) yielded similar findings, with even more pronounced temporal increases.

### 3.3 Multivariable Analysis

Female sex was associated with a slightly lower risk (RR, 0.93 95% CI, 0.91–0.94), in Medicaid only, with no association in MarketScan or CPRD (Table 2) and no overall association in pooled analyses. Compared with patients older than 13 years, children younger than 2 (1.97; 95% CI, 1.92–2.02) had a higher risk of presenting in DKA. When compared with White children, Black and Hispanic children had higher risks of presenting with DKA (1.14; 95% CI, 1.06–1.23 and 1.12; 95% CI, 1.10–1.15), with no differences observed for Asian or other racial groups. DKA risk increased incrementally over time relative to 2002–2005, rising in 2010–2015 (1.17; 95% CI, 1.03–1.33), 2016–2019 (1.31; 95% CI, 1.12–1.55) and the greatest increased risk in 2020 (1.60; 95% CI, 1.30–1.96). Higher social vulnerability was associated with increased risk, particularly in high‐vulnerability areas in CPRD (1.20; 95% CI, 1.02–1.40).

**TABLE 2:** Multivariable regression modelling risk of presenting in DKA at diagnosis of T1D, relative risks (95% CI).

	Medicaid 2002–2020	Medicaid 2010–2020^a^	MarketScan 2002–2020	CPRD 2002–2020	Pooled risk ratios^b^
Sociodemographic					
Female	0.93 (0.91, 0.94)^d^	0.92 (0.91, 0.94)^d^	1.02 (0.99, 1.05)	1.01 (0.95, 1.07)	0.98 (0.91, 1.06)
6 months < 2 years	1.95 (1.89, 2.01)^d^	1.85 (1.78, 1.92)^d^	1.99 (1.82, 2.10)^d^	2.12 (1.87, 2.40)^d^	1.97 (1.92, 2.02)^d^
2 < 7 years	1.22 (1.19, 1.25)^d^	1.19 (1.16, 1.22)^d^	1.02 (0.97, 1.07)	0.99 (0.91, 1.07)	1.07 (0.93, 1.25)
7–13 years	1.16 (1.14, 1.19)^d^	1.14 (1.11 1.16)^d^	1.14 (1.10, 1.18)^d^	0.90 (0.84, 0.96)	1.07 (0.96, 1.19)
> 13 years	Ref.	Ref.	Ref.	Ref.	Ref.
Race and ethnicity^c^					
White	Ref.		—	Ref.	
Black	1.16 (1.14, 1.19)^d^	1.11 (1.08, 1.14)^d^	—	1.06 (0.92, 1.22)	1.14 (1.06, 1.23)^d^
Hispanic	1.12 (1.10, 1.15)^d^	1.08 (1.06, 1.11)^d^	—	—	112 (1.10, 1.15)^d^
Asian	1.03 (0.96, 1.11)	1.03 (0.95, 1.11)	—	1.12 (0.99, 1.26)	1.06 (0.99, 1.14)
Other	0.98 (0.96, 1.00)	0.97 (0.94, 0.99)	—	1.00 (0.91, 1.10)	0.98 (0.96, 1.00)
Calendar year					
2002–2005	Ref.	—	Ref.	Ref.	
2006–2009	1.05 (1.02, 1.09)^d^	—	1.00 (0.94, 1.06)	1.13 (0.99, 1.29)	1.04 (0.99, 1.10)
2010–2015	1.12 (1.09, 1.16)^d^	Ref.	1.03 (0.98, 1.09)	1.46 (1.30, 1.64)^d^	1.17 (1.03, 1.33)^d^
2016–2019	1.26 (1.23, 1.30)^d^	1.14 (1.11, 1.16)^d^	1.10 (1.04, 1.16)^d^	1.69 (1.50, 1.91)^d^	1.31 (1.12, 1.55)^d^
2020	1.47 (1.42, 1.52)^d^	1.30 (1.27, 1.34)^d^	1.29 (1.20, 1.39)^d^	2.24 (1.96, 2.56)^d^	1.60 (1.30, 1.96)^d^
Social vulnerability					
Lowest tercile	—	Ref.	—	Ref.	
Middle tercile	—	1.05 (1.03, 1.07)^d^	—	1.09 (1.01, 1.17)^d^	1.05 (1.03, 1.08)^d^
Highest tercile	—	1.11 (1.08, 1.13)^d^	—	1.30 (1.21, 1.40)^d^	1.20 (1.02, 1.40)^d^

Abbreviations: CPRD: Clinical Practice Research Datalink; T1D: Type 1 diabetes.

^a^ Community level social vulnerability data were available for 2010–2020 for Medicaid.

^b^ Data were meta analysed using random effects meta‐analysis for Medicaid (2002–2020), MarketScan and CPRD. For race and ethnicity, data were pooled across Medicaid (2002–2020) and CPRD, as MarketScan does not contain information on race. For social vulnerability, data were pooled for Medicaid (2010–2020) and CPRD, as social vulnerability information was available in Medicaid after 2010.

^c^ Information on race, ethnicity and community‐level social vulnerability were available for Medicaid and CPRD, but not MarketScan. For CPRD, Hispanic patients were grouped into the other category.

^d^ Delineates estimates where the 95% confidence interval excludes 1.

## 4 Discussion

Evidence on population‐level trends in DKA at T1D diagnosis in the United States and England has been limited. Using three large, population‐based datasets, we observed that a significant number of children presented with DKA at diagnosis, with this proportion rising steadily over time across all cohorts, predating and following the COVID‐19 pandemic. Concurrently, the T1D incidence rose across all databases. In England, the proportion of youth presenting in DKA doubled over the study period, reaching levels similar to those in the United States by the end of follow‐up. The risk of DKA at diagnosis was higher among younger children and racial and ethnic minority groups.

We observed that the overall proportion of DKA at diagnosis was approximately 40% in the United States and 30% in England, aligning with rates reported in the SEARCH for Diabetes in Youth Study (31.1%–40.6%) [6, 15, 24], and a Welsh study (27.3%) [25]. Modest differences across studies likely reflect methodological heterogeneity and broader population representativeness of our data. In contrast to prospective cohorts such as TEDDY and DAISY, which report a proportion of DKA at diagnosis below 15% [17, 26], the higher proportions observed in our study likely reflect differences in surveillance intensity and participant composition in population‐based paediatric populations under routine care and limited diabetes education in the general population outside of these research study cohorts [27].

Consistent with prior studies, the proportion of children presenting with DKA at T1D diagnosis was highest among those younger than 2 years, potentially reflecting both difficulty recognising often nonspecific symptoms in very young children and more rapid metabolic decompensation, which may narrow the opportunity for diagnosis before DKA develops [28]. Black and Hispanic children also had higher risks of DKA. Although a single‐centre study reported a substantially stronger association among Black youth (odds ratio, 3.3) [29], the more modest associations observed in this study may be more generalizable as they were estimated using population‐based data spanning multiple geographic regions, health care systems and practice settings [30]. The higher risks among Black and Hispanic children, together with the association between greater neighbourhood social vulnerability and DKA, may reflect inequities in the timely recognition and evaluation of T1D. These could arise from reduced access to or continuity of primary care, symptoms being overlooked or attributed to other illnesses, failure to obtain glucose testing at an initial healthcare encounter or delays in referral and follow‐up [31, 32].

The mechanisms underpinning the increasing proportion of DKA at T1D diagnosis cannot be determined from this study, but are likely multifactorial. Delayed recognition of T1D symptoms, missed or delayed diagnostic evaluation, barriers to timely care and changes in the characteristics of children developing T1D may all contribute [33]. Secular increases in paediatric T1D incidence may also have increased the proportion of children without a first‐degree family history of T1D, whose families may be less familiar with its symptoms; however, family history was not reliably available in these data, and this explanation remains speculative [17, 34]. The pronounced increase in 2020 should be considered separately from the longer‐term trend and is consistent with international evidence of pandemic‐related disruptions in routine healthcare, as well as possible infection‐related inflammation or metabolic stress accelerating decompensation in susceptible children [35]. However, pandemic‐related changes in healthcare access, care‐seeking, coding and insurance enrolment may also have affected ascertainment of both incident T1D and DKA, particularly given the 6‐month continuous‐enrolment requirement. Accordingly, the 2020 estimates may reflect both underlying epidemiologic changes and pandemic‐related changes in healthcare utilisation and population enrolment. As 2020 was the terminal year of follow‐up, we could not assess whether these changes persisted beyond the initial pandemic period. The lower baseline proportion but steeper increase in CPRD may reflect differences between England and the United States in population composition, diagnostic pathways, ascertainment of DKA hospitalisations, as well as differences between claims and EHR data, although these explanations could not be evaluated directly [36]. Future qualitative and health systems research is needed to identify modifiable patient‐, clinician‐ and health‐system factors contributing to the rise in DKA at presentation.

Our study leveraged three nationally representative databases inclusive of underrepresented and socially vulnerable paediatric populations, providing highly generalizable estimates of DKA at diagnosis across the United States and England, with comprehensive coverage of both publicly and privately insured US children. These large population‐based datasets also enabled assessment of temporal trends across various patient subgroups. Study limitations are noted. First, the findings are generalizable to approximately 40% and 55% of US children who are covered by Medicaid or commercial insurance, but may not apply to 5% of US children without healthcare coverage. Second, incident T1D and DKA were identified using diagnosis and treatment‐based algorithms rather than paediatric‐specific validated clinical adjudication. DKA was ascertained using diagnosis codes without laboratory confirmation based on pH, anion gap, bicarbonate or serum ketone measurements. Accordingly, the findings reflect hospitalisations coded for DKA rather than biochemically confirmed events, and DKA severity could not be characterised. Although prior validation data indicated a PPV exceeding 94% for the T1D identification algorithm, residual misclassification remains possible, including the inclusion of children with Type 2, atypical or monogenic diabetes. Sensitivity analyses using a stricter insulin‐based definition ameliorate but do not entirely eliminate this concern [22]. Several potentially relevant factors, including family history, body mass index, immigration status, rurality, primary care access and symptom duration were unavailable or incompletely captured. Lastly, data on race and social vulnerability were lacking for commercially insured US children and for social vulnerability prior to 2010 among Medicaid recipients.

To our knowledge, this is the first cross‐national, population‐based analysis from the United States and England to characterise longitudinal trends in DKA at diagnosis across age, sex, race and social vulnerability, with generally increasing trends across subgroups, many evident before the COVID‐19 pandemic, and additional increases observed in 2020. These findings underscore worsening epidemiologic trends in DKA at diagnosis amid rising T1D incidence, highlighting a potentially preventable complication associated with substantial morbidity, mortality and long‐term adverse outcomes. This emphasises the need for further research to elucidate underlying drivers and identify effective strategies to mitigate severe presentation at diagnosis.

## Author Contributions

All authors were involved in the conception and design of the study, analysis and interpretation of data, critical revision of the article for important intellectual content and final approval of the article. C.V.D. and B.L.S. drafted the article, and C.V.D. provided administrative and logistical support and overall study supervision.

## Funding

This study was funded by Breakthrough T1D (3‐SRA‐2022‐1257‐S‐B).

## Conflicts of Interest

C.V.D. reports PI funding from NHLBI (R01HL163163) and NIDDK (R01DK139163). C.V.D. reports consulting fees from the US FDA, Takeda, Priovant, SpineBioPharma, AbyRx and the National evaluation system for health technology outside of the submitted study.

## Supporting information


**Appendix Figure 1A:** CONSORT flow diagrams.
**Appendix Figure 1B:** MarketScan.
**Appendix Figure 1C:** CPRD.
**Appendix Table 1:** Baseline characteristics of patients newly diagnosed with T1D by individual databases.
**Appendix Table 2:** Overall proportion of patients with DKA at diagnosis.
**Appendix Table 3:** Proportion of patients presenting in DKA by subgroups of sex and age.
**Appendix Table 4:** Proportion of patients presenting in DKA by subgroups of race and ethnicity, and SVI.
**Appendix Table 5:** Sensitivity analysis restricting to patients with an insulin prescription only.

## Data Availability

The data used in this study are proprietary third‐party data, not publicly available, and are used under licence for the current study.
