# Supplementary material for: Increasing Trends in Hospitalisation With Diabetic Ketoacidosis at Type 1 Diabetes Diagnosis Among Children and Youth in the United States and England, 2002–2020
Source: Diabetes Obes Metab. 2026 Aug 27;28(11):10615–24. doi: 10.1111/dom.71279 (PMC13646372; doi:10.1111/dom.71279)
Supplement: Supplementary file 1 [file DOM-28-10615-s001.docx]

Appendix Figure 1: CONSORT Flow diagrams

**Figure 1A: Medicaid**

Evidence of oral glucose lowering therapies during baseline or within 3 months after index (n = 16,558)

Insulin users: 0-21 yo

(n =366,626 )

(n = 203,799)

Not continuously enrolled 6 months prior to their first insulin fill (n = 162,827)

(n =98,119)

No diagnosis codes for T1D (n = 105,680)

Final Cohort

(n = 81,561)

**Appendix Figure 1B: MarketScan**

**Appendix Figure 1C:CPRD**

Abbreviations: UTS: Up to Standard; HES: Hospital Episode Statistics; SDI: Social Deprivation Index; T1D: Type 1 Diabetes

| **Appendix Table 1: Baseline characteristics of patients newly diagnosed with T1D by individual databases** | | | | |
| --- | --- | --- | --- | --- |
|  | **Overall Cohort**  **(n = 116,845)** | **Without DKA at diagnosis**  **(n = 68,554)** | **With DKA**  **at diagnosis**  **(n = 48,291)** | **SD ^b^** |
|  | **Medicaid Data ^a^** | | |  |
|  | **(n = 81,561)** | **(n = 46,835)** | **(n = 34,726)** |  |
| **Sociodemographics** |  |  |  |  |
| Age, mean (SD), y | 10.2 (4.9) | 10.5 (4.8) | 9.7 (5.0) | -16.3 |
| <2y | 2,746 (3.4) | 841 (1.8) | 1,905 (5.5) | 19.8 |
| 2 -<7y | 17,361 (21.3) | 9,711 (20.7) | 7,650 (22.0) | 3.2 |
| 7-13y | 40,231 (49.3) | 22,975 (49.1) | 17,256 (49.7) | 1.2 |
| >13y | 21,223 (26.0) | 13,308 (28.4) | 7,915 (22.8) | -12.9 |
| Female | 38,978 (47.8) | 23,003 (49.1) | 15,975 (46.0) | -6.2 |
| **Race and Ethnicity** |  |  |  |  |
| White | 35,710 (43.8) | 21,323 (45.5) | 14,387 (41.4) | -8.3 |
| Black | 17,575 (21.6) | 9,547 (20.4) | 8,028 (23.1) | 6.5 |
| Hispanic | 14,113 (17.3) | 7,700 (16.4) | 6,413 (18.5) | 5.5 |
| Asian | 1,073 (1.3) | 626 (1.3) | 447 (1.3) | 0.0 |
| Other | 13,090 (16.1) | 7,639 (16.3) | 5,451 (15.7) | -1.6 |
| **Calendar Year** |  |  |  |  |
| 2002 – 2005 | 9,587 (11.8) | 6,069 (13.0) | 3,518 (10.1) | -9.1 |
| 2006 – 2009 | 11,227 (13.8) | 6,893 (14.7) | 4,334 (12.5) | -6.4 |
| 2010 – 2015 | 27,147 (33.3) | 16,092 (34.4) | 11,055 (31.8) | -5.5 |
| 2016 – 2019 | 25,395 (31.1) | 13,879 (29.6) | 11,516 (33.2) | -7.8 |
| 2020 | 8,205 (10.1) | 3,902 (8.3) | 4,303 (12.4) | -13.5 |
| **Social vulnerability** |  |  |  |  |
| Lowest tercile | 19,921 (34.5) | 11,653 (36.1) | 8,268 (32.4) | -7.8 |
| Middle tercile | 18,174 (31.4) | 10,215 (31.7) | 7,959 (31.2) | -1.1 |
| Highest tercile | 19,726 (34.1) | 10,408 (32.3) | 9,318 (36.5) | 8.8 |
|  | **MarketScan Data ^a^** | | |  |
| **Sociodemographics** | **(n = 24,176)** | **(n = 13,812)** | **(n = 10,364)** |  |
| Age, mean (SD), y | 10.8 (5.0) | 11.0 (4.9) | 10.6 (5.1) | -8.0 |
| <2y | 583 (2.4) | 129 (0.9) | 454 (4.4) | 21.9 |
| 2 -<7y | 4,487 (18.6) | 2,703 (19.6) | 1,784 (17.2) | -6.2 |
| 7-13y | 11,890 (49.2) | 6,593 (47.7) | 5,297 (51.1) | 6.8 |
| >13y | 7,216 (29.9) | 4,387 (31.8) | 2,829 (27.3) | -9.9 |
| Female | 10,785 (44.6) | 6,099 (44.2) | 4,686 (45.2) | 2.0 |
| **Calendar Year** |  |  |  |  |
| 2002 – 2005 | 2,235 (9.2) | 1,313 (9.5) | 922 (8.9) | -2.1 |
| 2006 – 2009 | 4,800 (19.9) | 2,839 (20.6) | 1,961 (18.9) | -4.3 |
| 2010 – 2015 | 10,814 (44.7) | 6,266 (45.4) | 4,548 (43.9) | -3.0 |
| 2016 – 2019 | 5,072 (21.0) | 2,803 (20.3) | 2,269 (21.9) | -3.9 |
| 2020 | 1,255 (5.2) | 591 (4.3) | 664 (6.4) | -9.3 |
|  | **CPRD Data ^a^** | | |  |
| **Sociodemographics** | **(n = 11,108)** | **(n = 7,907)** | **(n = 3,201)** |  |
| Age, mean (SD), y | 10.9 (5.0) | 10.9 (4.9) | 10.8 (5.4) | -1.9 |
| <2y | 188 (1.7) | 72 (0.9) | 116 (3.6) | 18.3 |
| 2 -<7y | 2,206 (19.9) | 1,562 (19.8) | 644 (20.1) | 0.8 |
| 7-13y | 5,269 (47.4) | 3,853 (48.7) | 1,416 (44.2) | -9.0 |
| >13y | 3,445 (31.0) | 2,420 (30.6) | 1,025 (32.0) | 3.0 |
| Female | 4,920 (44.3) | 3,494 (44.2) | 1,426 (44.6) | 0.8 |
| **Race and Ethnicity** |  |  |  |  |
| White | 9,173 (82.6) | 6,603 (83.5) | 2,570 (80.3) | -8.3 |
| Black | 421 (3.8) | 276 (3.5) | 145 (4.5) | 5.1 |
| Hispanic | 0 | 0 | 0 |  |
| Asian | 565 (5.1) | 372 (4.7) | 193 (6.0) | 5.8 |
| Other | 949 (8.5) | 656 (8.3) | 293 (9.2) | 3.2 |
| **Calendar Year** |  |  |  |  |
| 2002 – 2005 | 1,335 (12.0) | 1,068 (13.5) | 267 (8.3) | -16.7 |
| 2006 – 2009 | 2,182 (19.6) | 1,694 (21.4) | 488 (15.3) | -15.8 |
| 2010 – 2015 | 4,066 (36.6) | 2,887 (36.5) | 1,179 (36.8) | 0.6 |
| 2016 – 2019 | 2,734 (24.6) | 1,816 (23.0) | 918 (28.7) | -13.4 |
| 2020 | 791 (7.1) | 442 (5.6) | 349 (10.9) | -19.4 |
| **Social vulnerability** |  |  |  |  |
| Lowest tercile | 3,235 (29.1) | 2,419 (30.6) | 816 (25.5) | -11.4 |
| Middle tercile | 4,266 (38.4) | 3,086 (39.0) | 1,180 (36.9) | -4.3 |
| Highest tercile | 3,607 (32.5) | 2,402 (30.4) | 1,205 (37.6) | 15.2 |
| Abbreviations: T1D: Type 1 diabetes; SD: Standardized Differences  * Information on race, ethnicity, and community-level social vulnerability were available for Medicaid (after 2010 for social vulnerability) and CPRD, but not MarketScan. The denominator for social vulnerability estimates in Medicaid was 57,821 (25,545 with DKA and 32,276 without) | | | | |

| **Appendix Table 2: Overall proportion of patients with DKA at diagnosis** | | | |
| --- | --- | --- | --- |
|  | Medicaid (US)  N = 81,561 | MarketScan (US)  N = 24,176 | CPRD (UK)  N = 11,108 |
| **Year** |  |  |  |
| 2002 | 34.4 (32.4, 36.4) | 42.0 (36.7, 47.6) | 17.9 (13.3, 23.7) |
| 2003 | 35.3 (33.4, 37.2) | 42.2 (38.1, 46.3) | 18.1 (14.0, 23.0) |
| 2004 | 37.1 (35.2, 39.0) | 41.5 (37.8, 45.3) | 21.9 (18.1, 26.2) |
| 2005 | 39.6 (37.7, 41.5) | 39.9 (36.4, 43.6) | 20.5 (17.1, 24.5) |
| 2006 | 39.3 (37.4, 41.2) | 38.1 (34.3, 42.1) | 24.6 (21.0, 28.6) |
| 2007 | 39.6 (37.7, 41.4) | 40.5 (37.3, 43.9) | 22.1 (18.8, 25.9) |
| 2008 | 38.5 (36.7, 40.4) | 42.3 (39.7, 44.9) | 21.3 (18.1, 24.9) |
| 2009 | 37.4 (35.7, 39.0) | 40.8 (38.7, 43.0) | 21.7 (18.6, 25.2) |
| 2010 | 38.4 (36.8, 40.1) | 39.0 (36.8, 41.3) | 26.4 (23.1, 30.0) |
| 2011 | 39.7 (38.2, 41.2) | 41.7 (39.6, 43.8) | 27.5 (24.2, 31.1) |
| 2012 | 41.1 (39.6, 42.6) | 41.2 (39.1, 43.3) | 25.6 (22.4, 29.0) |
| 2013 | 40.0 (38.6, 41.4) | 42.3 (39.9, 44.7) | 35.4 (32.0, 39.0) |
| 2014 | 41.0 (39.6, 42.3) | 44.9 (42.6, 47.3) | 27.9 (24.7, 31.3) |
| 2015 | 43.0 (41.7, 44.3) | 44.3 (41.7, 46.9) | 30.6 (27.3, 34.1) |
| 2016 | 42.2 (40.9, 43.4) | 43.1 (40.5, 45.7) | 28.4 (25.2, 31.7) |
| 2017 | 45.8 (44.6, 47.0) | 46.4 (43.7, 49.2) | 32.8 (29.4, 36.4) |
| 2018 | 47.8 (46.6, 49.0) | 44.9 (42.1, 47.7) | 37.4 (33.7, 41.2) |
| 2019 | 45.5 (44.2, 46.7) | 44.8 (42.0, 47.6) | 36.5 (33.0, 40.2) |
| 2020 | 52.4 (51.4, 53.5) | 52.9 (50.1, 55.7) | 44.1 (40.7, 47.6) |
| **P-value for trend** | <.0001 | 0.0007 | <.0001 |
| **P-value for trend through 2019** | <.0001 | 0.0016 | <.0001 |

| **Appendix Table 3: Proportion of patients presenting in DKA by subgroups of sex and age** | | | |
| --- | --- | --- | --- |
|  | **Medicaid** | **MarketScan** | **CPRD** |
| **Male** |  |  |  |
| 2002 | 34.8 (31.9, 37.7) | 44.9 (37.7, 52.3) | 14.2 (8.9, 21.8) |
| 2003 | 38.0 (35.3, 40.8) | 46.0 (40.3, 51.8) | 17.6 (12.5, 24.3) |
| 2004 | 39.0 (36.4, 41.8) | 39.8 (34.9, 44.9) | 23.5 (18.5, 29.3) |
| 2005 | 40.8 (38.1, 43.5) | 39.1 (34.2, 44.2) | 17.9 (13.8, 23.0) |
| 2006 | 39.5 (36.9, 42.2) | 38.8 (33.7, 44.1) | 24.5 (19.7, 29.9) |
| 2007 | 39.6 (37.0, 42.3) | 41.1 (36.8, 45.5) | 22.2 (17.9, 27.2) |
| 2008 | 40.2 (37.6, 42.8) | 40.2 (36.8, 43.8) | 23.6 (19.4, 28.5) |
| 2009 | 37.5 (35.2, 39.9) | 38.3 (35.4, 41.2) | 22.2 (18.1, 27.0) |
| 2010 | 39.7 (37.4, 42.0) | 39.0 (36.0, 42.0) | 26.3 (21.9, 31.3) |
| 2011 | 40.3 (38.2, 42.4) | 41.0 (38.2, 43.9) | 23.1 (19.0, 27.8) |
| 2012 | 42.9 (40.8, 45.0) | 40.7 (37.9, 43.5) | 27.5 (23.1, 32.3) |
| 2013 | 41.1 (39.2, 43.1) | 39.5 (36.4, 42.7) | 34.8 (30.3, 39.5) |
| 2014 | 41.8 (39.9, 43.6) | 46.0 (42.9, 49.2) | 29.1 (24.7, 33.9) |
| 2015 | 44.2 (42.5, 46.0) | 43.0 (39.5, 46.6) | 30.4 (26.0, 35.1) |
| 2016 | 43.3 (41.6, 45.0) | 43.2 (39.7, 46.7) | 26.2 (22.1, 30.6) |
| 2017 | 47.5 (45.8, 49.2) | 44.6 (41.0, 48.3) | 30.5 (26.2, 35.3) |
| 2018 | 49.3 (47.6, 50.9) | 45.3 (41.6, 49.0) | 36.9 (31.8, 42.3) |
| 2019 | 47.2 (45.5, 48.8) | 46.1 (42.4, 50.0) | 35.7 (31.1, 40.6) |
| 2020 | 54.4 (52.9, 55.9) | 53.4 (49.7, 57.0) | 49.1 (44.5, 53.7) |
| **P-value for trend** | <.0001 | 0.0220 | <.0001 |
| **P-value for trend through 2019** | <.0001 | 0.1181 | <.0001 |
| **Female** |  |  |  |
| 2002 | 34.0 (31.2, 36.9) | 38.4 (30.7, 46.7) | 22.3 (15.1, 31.8) |
| 2003 | 32.5 (29.9, 35.2) | 37.9 (32.3, 44.0) | 18.6 (12.6, 26.6) |
| 2004 | 35.2 (32.6, 37.9) | 43.6 (38.1, 49.2) | 19.5 (14.2, 26.2) |
| 2005 | 38.3 (35.7, 41.1) | 40.9 (35.8, 46.2) | 24.1 (18.6, 30.6) |
| 2006 | 39.0 (36.3, 41.8) | 37.3 (31.6, 43.3) | 24.8 (19.5, 30.9) |
| 2007 | 39.5 (37.0, 42.1) | 39.8 (34.9, 44.9) | 22.1 (17.0, 28.1) |
| 2008 | 36.8 (34.3, 39.5) | 44.8 (40.9, 48.8) | 18.1 (13.7, 23.4) |
| 2009 | 37.2 (34.9, 39.6) | 43.7 (40.6, 46.9) | 21.2 (16.7, 26.4) |
| 2010 | 37.2 (34.9, 39.5) | 39.1 (35.8, 42.5) | 26.5 (21.7, 31.9) |
| 2011 | 39.1 (37.0, 41.3) | 42.6 (39.4, 45.7) | 32.7 (27.5, 38.2) |
| 2012 | 39.3 (37.1, 41.4) | 41.8 (38.7, 44.9) | 23.3 (19.0, 28.3) |
| 2013 | 38.8 (36.8, 40.8) | 45.9 (42.2, 49.5) | 36.2 (31.0, 41.7) |
| 2014 | 40.0 (38.1, 42.0) | 43.6 (40.1, 47.1) | 26.4 (21.9, 31.5) |
| 2015 | 41.5 (39.6, 43.4) | 45.8 (41.9, 49.8) | 30.8 (26.0, 36.1) |
| 2016 | 40.9 (39.1, 42.7) | 42.9 (39.1, 46.8) | 31.1 (26.4, 36.3) |
| 2017 | 43.8 (42.1, 45.6) | 48.8 (44.6, 53.0) | 35.9 (30.7, 41.5) |
| 2018 | 46.1 (44.3, 47.9) | 44.3 (40.1, 48.6) | 37.9 (32.7, 43.4) |
| 2019 | 43.4 (41.6, 45.2) | 43.0 (38.9, 47.3) | 37.6 (32.1, 43.4) |
| 2020 | 50.1 (48.5, 51.7) | 52.3 (48.1, 56.5) | 37.5 (32.6, 42.8) |
| **P-value for trend** | <.0001 | 0.0005 | <.0001 |
| **P-value for trend through 2019** | <.0001 | 0.0027 | <.0001 |
| **Age 6m-<2** |  |  |  |
| 2002-2004 | 67.8 (62.7, 72.5) | 68.4 (55.5, 79.0) | 33.3 (15.2, 58.3) |
| 2005-2006 | 66.5 (60.1, 72.4) | 88.2 (73.4, 95.3) | 57.7 (38.9, 74.5) |
| 2007-2008 | 69.1 (62.7, 74.9) | 67.9 (54.5, 78.9) | 76.9 (49.7, 91.8) |
| 2009-2010 | 69.3 (63.2, 74.8) | 73.7 (64.3, 81.4) | 61.9 (40.9, 79.2) |
| 2011-2012 | 67.2 (61.7, 72.3) | 79.3 (69.3, 86.6) | 57.1 (39.1, 73.5) |
| 2013-2014 | 71.3 (66.0, 76.1) | 80.3 (69.6, 87.9) | 66.7 (47.8, 81.4) |
| 2015-2016 | 71.8 (66.9, 76.2) | 80.0 (68.7, 87.9) | 73.7 (51.2, 88.2) |
| 2017-2018 | 72.7 (68.1, 76.8) | 84.7 (73.5, 91.8) | 60.9 (40.8, 77.8) |
| 2019-2020 | 67.0 (62.1, 71.6) | 82.5 (71.4, 90.0) | 68.8 (44.4, 85.8) |
| **P-value for trend** | 0.2172 | 0.1704 | 0.1360 |
| **Age 2-<7** |  |  |  |
| 2002 | 38.5 (33.9, 43.3) | 31.6 (21.0, 44.5) | 24.3 (13.4, 40.1) |
| 2003 | 37.0 (33.0, 41.3) | 42.9 (34.6, 51.6) | 8.1 (3.5, 17.5) |
| 2004 | 39.0 (35.2, 42.9) | 42.8 (35.5, 50.4) | 18.3 (11.7, 27.3) |
| 2005 | 41.9 (38.1, 45.8) | 33.3 (26.2, 41.4) | 23.0 (15.4, 32.9) |
| 2006 | 40.3 (36.4, 44.2) | 40.9 (32.8, 49.6) | 27.6 (20.0, 36.8) |
| 2007 | 42.6 (38.7, 46.5) | 33.8 (26.6, 41.8) | 25.0 (17.7, 34.1) |
| 2008 | 43.1 (39.2, 47.0) | 38.6 (33.0, 44.5) | 22.8 (16.1, 31.3) |
| 2009 | 39.2 (35.8, 42.6) | 37.4 (32.9, 42.1) | 28.1 (20.9, 36.7) |
| 2010 | 40.2 (36.8, 43.7) | 32.0 (27.3, 37.1) | 27.7 (20.5, 36.4) |
| 2011 | 42.0 (38.8, 45.2) | 40.2 (35.5, 45.1) | 27.1 (20.5, 35.0) |
| 2012 | 44.0 (40.9, 47.1) | 40.9 (36.1, 45.9) | 32.6 (25.2, 41.0) |
| 2013 | 45.4 (42.5, 48.4) | 38.7 (33.2, 44.5) | 35.5 (28.7, 43.1) |
| 2014 | 44.1 (41.2, 47.1) | 38.0 (32.8, 43.4) | 20.1 (14.3, 27.6) |
| 2015 | 43.1 (40.2, 46.0) | 42.5 (36.5, 48.7) | 29.5 (22.5, 37.5) |
| 2016 | 44.6 (41.8, 47.4) | 42.7 (36.5, 49.2) | 35.0 (27.6, 43.2) |
| 2017 | 47.5 (44.8, 50.3) | 45.2 (38.7, 51.8) | 31.7 (24.1, 40.4) |
| 2018 | 47.4 (44.6, 50.1) | 44.0 (37.6, 50.7) | 41.0 (32.7, 49.9) |
| 2019 | 46.6 (44.0, 49.3) | 45.2 (38.3, 52.4) | 34.9 (27.2, 43.4) |
| 2020 | 50.7 (48.1, 53.2) | 44.4 (37.8, 51.3) | 39.6 (31.7, 48.0) |
| **P-value for trend** | <.0001 | 0.0069 | <.0001 |
| **P-value for trend through 2019** | <.0001 | 0.0158 | 0.0003 |
| **Age: 7-13** |  |  |  |
| 2002 | 35.6 (32.6, 38.7) | 43.3 (35.7, 51.3) | 17.3 (11.2, 25.7) |
| 2003 | 34.8 (32.0, 37.7) | 46.6 (40.6, 52.8) | 21.3 (15.0, 29.2) |
| 2004 | 37.4 (34.7, 40.3) | 42.3 (36.9, 47.8) | 21.2 (15.7, 27.9) |
| 2005 | 39.0 (36.3, 41.9) | 43.7 (38.6, 48.9) | 16.1 (11.6, 21.9) |
| 2006 | 39.0 (36.2, 41.9) | 34.8 (29.6, 40.5) | 22.2 (17.4, 28.0) |
| 2007 | 39.7 (37.1, 42.3) | 42.2 (37.7, 46.9) | 18.8 (14.4, 24.1) |
| 2008 | 38.7 (36.0, 41.4) | 44.6 (40.9, 48.4) | 19.8 (15.6, 24.8) |
| 2009 | 37.4 (35.1, 39.8) | 42.5 (39.6, 45.5) | 18.9 (14.7, 24.0) |
| 2010 | 37.9 (35.7, 40.2) | 42.2 (39.1, 45.4) | 24.2 (19.7, 29.3) |
| 2011 | 39.0 (36.9, 41.2) | 42.5 (39.5, 45.5) | 28.6 (23.8, 33.9) |
| 2012 | 41.9 (39.7, 44.1) | 43.8 (40.8, 46.8) | 22.6 (18.4, 27.5) |
| 2013 | 38.5 (36.6, 40.5) | 43.7 (40.4, 47.1) | 33.3 (28.3, 38.8) |
| 2014 | 41.7 (39.8, 43.6) | 47.7 (44.3, 51.2) | 28.5 (23.9, 33.6) |
| 2015 | 43.8 (42.0, 45.6) | 47.2 (43.3, 51.0) | 27.6 (23.1, 32.6) |
| 2016 | 42.2 (40.4, 43.9) | 44.8 (41.1, 48.6) | 24.3 (20.3, 28.9) |
| 2017 | 46.5 (44.7, 48.2) | 48.1 (44.1, 52.1) | 31.7 (26.9, 36.8) |
| 2018 | 47.7 (46.0, 49.3) | 44.5 (40.5, 48.5) | 32.3 (27.3, 37.8) |
| 2019 | 45.7 (44.0, 47.4) | 45.4 (41.4, 49.5) | 33.2 (28.3, 38.5) |
| 2020 | 53.5 (52.0, 55.0) | 53.9 (49.9, 57.9) | 43.6 (38.8, 48.5) |
| **P-value for trend** | <.0001 | 0.0120 | <.0001 |
| **P-value for trend through 2019** | <.0001 | 0.0631 | <.0001 |
| **Age >13** |  |  |  |
| 2002 | 25.5 (22.3, 29.0) | 40.9 (31.4, 51.0) | 15.4 (8.6, 26.1) |
| 2003 | 28.4 (25.2, 31.9) | 30.6 (23.7, 38.5) | 18.8 (11.9, 28.4) |
| 2004 | 28.5 (25.2, 32.1) | 35.9 (29.1, 43.3) | 25.0 (18.2, 33.3) |
| 2005 | 33.6 (30.1, 37.3) | 33.0 (26.7, 40.0) | 20.3 (14.7, 27.2) |
| 2006 | 34.3 (30.8, 37.9) | 37.3 (30.0, 45.1) | 24.6 (18.3, 32.3) |
| 2007 | 31.8 (28.5, 35.4) | 40.4 (34.4, 46.7) | 22.8 (16.9, 29.9) |
| 2008 | 30.1 (26.8, 33.5) | 37.8 (33.1, 42.7) | 21.1 (15.6, 27.9) |
| 2009 | 30.5 (27.4, 33.8) | 36.3 (32.2, 40.7) | 19.7 (14.8, 25.8) |
| 2010 | 33.4 (30.3, 36.7) | 34.9 (30.9, 39.2) | 27.1 (21.3, 33.8) |
| 2011 | 34.1 (31.1, 37.2) | 37.8 (34.0, 41.8) | 23.2 (17.7, 29.9) |
| 2012 | 32.8 (29.9, 35.9) | 35.6 (32.0, 39.3) | 23.9 (18.6, 30.2) |
| 2013 | 33.5 (30.8, 36.2) | 39.3 (35.2, 43.6) | 36.0 (30.2, 42.3) |
| 2014 | 33.8 (31.4, 36.4) | 42.5 (38.4, 46.6) | 29.4 (23.7, 35.9) |
| 2015 | 37.9 (35.5, 40.4) | 37.9 (33.5, 42.5) | 33.5 (27.6, 39.9) |
| 2016 | 37.2 (34.9, 39.6) | 38.8 (34.5, 43.3) | 29.5 (23.9, 35.9) |
| 2017 | 40.1 (37.8, 42.4) | 41.6 (37.0, 46.5) | 33.8 (27.8, 40.3) |
| 2018 | 45.4 (43.0, 47.8) | 43.3 (38.4, 48.2) | 41.6 (35.0, 48.6) |
| 2019 | 41.8 (39.5, 44.2) | 42.1 (37.4, 46.9) | 41.1 (34.7, 47.8) |
| 2020 | 50.4 (48.4, 52.4) | 52.2 (47.4, 56.9) | 46.8 (40.7, 53.0) |
| **P-value for trend** | <.0001 | 0.0016 | <.0001 |
| **P-value for trend through 2019** | <.0001 | 0.0070 | <.0001 |

| **Appendix Table 4: Proportion of patients presenting in DKA by subgroups of race and ethnicity, and SVI** | | | |
| --- | --- | --- | --- |
|  | **Medicaid** | **CPRD** |  |
| **Racial and ethnic minorities** |  |  |  |
| 2002 | 36.8 (33.9, 39.7) | 40.9 (23.3, 61.3) |  |
| 2003 | 39.7 (37.0, 42.5) | 27.3 (13.2, 48.2) |  |
| 2004 | 40.4 (37.7, 43.2) | 22.9 (13.3, 36.5) |  |
| 2005 | 41.9 (39.1, 44.6) | 23.8 (15.0, 35.6) |  |
| 2006 | 41.0 (38.4, 43.7) | 28.6 (18.9, 40.7) |  |
| 2007 | 41.2 (38.6, 43.8) | 30.1 (20.8, 41.4) |  |
| 2008 | 41.2 (38.6, 43.8) | 25.0 (16.6, 35.8) |  |
| 2009 | 38.7 (36.5, 41.0) | 27.0 (18.8, 37.0) |  |
| 2010 | 41.3 (39.1, 43.5) | 28.7 (20.3, 39.0) |  |
| 2011 | 41.8 (39.7, 43.9) | 28.8 (21.2, 37.9) |  |
| 2012 | 40.6 (38.5, 42.7) | 23.4 (16.4, 32.2) |  |
| 2013 | 41.0 (39.1, 42.9) | 29.1 (21.6, 37.8) |  |
| 2014 | 43.3 (41.5, 45.1) | 27.4 (20.1, 36.1) |  |
| 2015 | 43.4 (41.7, 45.1) | 30.8 (24.1, 38.4) |  |
| 2016 | 43.8 (42.2, 45.4) | 35.9 (28.5, 43.9) |  |
| 2017 | 46.4 (44.9, 48.0) | 37.3 (30.0, 45.3) |  |
| 2018 | 48.4 (46.8, 49.9) | 38.3 (30.7, 46.5) |  |
| 2019 | 46.9 (45.3, 48.5) | 37.5 (30.0, 45.6) |  |
| 2020 | 53.5 (52.2, 54.9) | 46.6 (39.9, 53.4) |  |
| **P-value for trend** | <.0001 | 0.0144 |  |
| **P-value for trend through 2019** | <.0001 | 0.0706 |  |
| **White patients** |  |  |  |
| 2002 | 32.0 (29.3, 34.9) | 15.1 (10.7, 21.0) |  |
| 2003 | 30.9 (28.4, 33.5) | 17.3 (13.1, 22.4) |  |
| 2004 | 33.9 (31.3, 36.5) | 21.7 (17.7, 26.3) |  |
| 2005 | 37.4 (34.8, 40.0) | 20.0 (16.3, 24.3) |  |
| 2006 | 37.4 (34.8, 40.2) | 24.0 (20.2, 28.3) |  |
| 2007 | 37.9 (35.3, 40.5) | 20.8 (17.3, 24.8) |  |
| 2008 | 35.8 (33.2, 38.4) | 20.7 (17.4, 24.5) |  |
| 2009 | 35.8 (33.5, 38.2) | 20.8 (17.6, 24.6) |  |
| 2010 | 35.0 (32.7, 37.4) | 26.0 (22.5, 29.9) |  |
| 2011 | 37.3 (35.2, 39.5) | 27.2 (23.6, 31.1) |  |
| 2012 | 41.7 (39.5, 44.0) | 26.0 (22.5, 29.7) |  |
| 2013 | 38.8 (36.8, 40.9) | 36.6 (32.9, 40.6) |  |
| 2014 | 38.2 (36.2, 40.1) | 28.0 (24.5, 31.7) |  |
| 2015 | 42.4 (40.4, 44.3) | 30.5 (26.8, 34.5) |  |
| 2016 | 39.8 (37.9, 41.8) | 26.5 (23.2, 30.2) |  |
| 2017 | 44.8 (42.8, 46.7) | 31.6 (27.8, 35.6) |  |
| 2018 | 46.9 (44.9, 48.8) | 37.1 (33.0, 41.5) |  |
| 2019 | 43.4 (41.5, 45.3) | 36.2 (32.3, 40.4) |  |
| 2020 | 50.6 (48.9, 52.4) | 43.3 (39.3, 47.3) |  |
| **P-value for trend** | <.0001 | <.0001 |  |
| **P-value for trend through 2019** | <.0001 | <.0001 |  |
| **High social vulnerability** |  |  |  |
| 2002 |  | 22.7 (14.3, 34.2) |  |
| 2003 |  | 16.9 (10.3, 26.3) |  |
| 2004 |  | 23.8 (17.1, 32.1) |  |
| 2005 |  | 23.7 (17.9, 30.6) |  |
| 2006 |  | 30.7 (24.4, 37.8) |  |
| 2007 |  | 30.2 (23.8, 37.5) |  |
| 2008 |  | 26.3 (20.3, 33.5) |  |
| 2009 |  | 26.6 (20.8, 33.2) |  |
| 2010 | 43.0 (40.2, 45.8) | 30.4 (24.4, 37.2) |  |
| 2011 | 46.2 (43.5, 48.8) | 33.7 (27.3, 40.7) |  |
| 2012 | 45.7 (43.1, 48.4) | 24.8 (19.6, 30.8) |  |
| 2013 | 43.1 (40.6, 45.5) | 43.1 (36.5, 49.8) |  |
| 2014 | 41.6 (39.1, 44.3) | 32.6 (26.9, 38.9) |  |
| 2015 | 43.4 (41.0, 45.7) | 36.1 (30.3, 42.4) |  |
| 2016 | 41.9 (39.6, 44.2) | 34.0 (28.5, 39.9) |  |
| 2017 | 46.9 (44.7, 49.2) | 39.7 (33.8, 46.1) |  |
| 2018 | 50.8 (48.6, 53.0) | 41.8 (35.3, 48.6) |  |
| 2019 | 49.3 (47.0, 51.5) | 40.2 (33.9, 46.8) |  |
| 2020 | 55.2 (53.6, 56.8) | 47.8 (41.6, 54.0) |  |
| **P-value for trend** | 0.0194 | <.0001 |  |
| **P-value for trend through 2019** | 0.1033 | <.0001 |  |
| **Low social vulnerability** |  |  |  |
| 2002 |  | 18.2 (10.2, 30.3) |  |
| 2003 |  | 11.4 (6.1, 20.3) |  |
| 2004 |  | 23.3 (16.9, 31.2) |  |
| 2005 |  | 21.7 (14.9, 30.5) |  |
| 2006 |  | 18.9 (13.3, 26.1) |  |
| 2007 |  | 19.7 (13.9, 27.2) |  |
| 2008 |  | 16.2 (11.6, 22.2) |  |
| 2009 |  | 17.2 (12.4, 23.5) |  |
| 2010 | 34.7 (31.9, 37.6) | 21.9 (16.6, 28.2) |  |
| 2011 | 36.5 (34.0, 39.1) | 22.9 (17.5, 29.4) |  |
| 2012 | 37.7 (35.0, 40.3) | 23.6 (18.3, 29.9) |  |
| 2013 | 37.4 (35.1, 39.7) | 35.5 (29.4, 42.1) |  |
| 2014 | 37.6 (35.3, 39.9) | 27.3 (21.5, 34.0) |  |
| 2015 | 40.8 (38.6, 43.2) | 24.4 (19.0, 30.7) |  |
| 2016 | 39.5 (37.4, 41.6) | 21.4 (16.3, 27.5) |  |
| 2017 | 45.6 (43.4, 47.8) | 27.9 (22.3, 34.1) |  |
| 2018 | 44.5 (42.5, 46.6) | 34.6 (28.2, 41.5) |  |
| 2019 | 43.6 (41.6, 45.6) | 30.4 (24.2, 37.4) |  |
| 2020 | 49.0 (47.0, 50.9) | 40.0 (33.8, 46.6) |  |
| **P-value for trend** | <.0001 | <.0001 |  |
| **P-value for trend through 2019** | 0.0002 | 0.0005 |  |

| **Appendix Table 5: Sensitivity analysis restricting to patients with an insulin prescription only** | | | |
| --- | --- | --- | --- |
|  | Medicaid | MarketScan | CPRD |
| **Year** |  |  |  |
| 2002 | 17.4 (16.3, 18.6) | 23.8 (20.4, 27.5) | 6.0 (4.4, 8.2) |
| 2003 | 19.9 (18.7, 21.1) | 26.3 (23.5, 29.3) | 6.4 (4.9, 8.3) |
| 2004 | 21.4 (20.2, 22.6) | 28.1 (25.4, 30.9) | 10.0 (8.2, 12.2) |
| 2005 | 23.4 (22.2, 24.7) | 28.2 (25.5, 31.0) | 10.1 (8.3, 12.2) |
| 2006 | 22.4 (21.2, 23.7) | 26.2 (23.3, 29.2) | 15.7 (13.3, 18.5) |
| 2007 | 24.3 (23.1, 25.6) | 25.9 (23.6, 28.3) | 15.5 (13.0, 18.3) |
| 2008 | 22.2 (21.0, 23.4) | 23.5 (21.8, 25.2) | 16.7 (14.2, 19.6) |
| 2009 | 24.2 (23.0, 25.4) | 29.0 (27.3, 30.6) | 17.5 (14.9, 20.3) |
| 2010 | 22.4 (21.3, 23.4) | 28.2 (26.5, 29.9) | 21.2 (18.5, 24.3) |
| 2011 | 25.6 (24.6, 26.7) | 29.7 (28.1, 31.4) | 23.0 (20.2, 26.1) |
| 2012 | 27.2 (26.1, 28.3) | 30.4 (28.7, 32.1) | 20.8 (18.1, 23.7) |
| 2013 | 22.0 (21.1, 22.8) | 30.5 (28.6, 32.4) | 29.7 (26.7, 32.8) |
| 2014 | 27.6 (26.6, 28.6) | 33.1 (31.2, 35.1) | 23.5 (20.7, 26.5) |
| 2015 | 30.5 (29.5, 31.6) | 31.6 (29.5, 33.7) | 26.4 (23.5, 29.5) |
| 2016 | 28.3 (27.4, 29.2) | 32.7 (30.6, 34.9) | 25.1 (22.3, 28.2) |
| 2017 | 31.6 (30.6, 32.5) | 33.4 (31.3, 35.7) | 28.7 (25.7, 32.0) |
| 2018 | 35.8 (34.8, 36.8) | 32.2 (30.0, 34.5) | 33.1 (29.7, 36.6) |
| 2019 | 35.5 (34.5, 36.6) | 32.6 (30.3, 34.9) | 31.7 (28.5, 35.1) |
| 2020 | 43.5 (42.6, 44.5) | 40.8 (38.4, 43.2) | 40.3 (37.1, 43.6) |
| **P-value for trend** | <.0001 | <.0001 | <.0001 |
| **P-value for trend through 2019** | <.0001 | <.0001 | <.0001 |
